# Exploring How Older Adults Use a Smart Speaker–Based Voice Assistant in Their First Interactions: Qualitative Study

**DOI:** 10.2196/20427

**Published:** 2021-01-13

**Authors:** Sunyoung Kim

**Affiliations:** 1 School of Communication and Information Rutgers University New Brunswick, NJ United States

**Keywords:** older adults, voice assistant, smart speaker, technology acceptance, quality of life

## Abstract

**Background:**

Smart speaker–based voice assistants promise support for the aging population, with the advantages of hands-free and eyes-free interaction modalities to handle requests. However, little is known about how older adults perceive the benefits of this type of device.

**Objective:**

This study investigates how older adults experience and respond to a voice assistant when they first interact with it. Because first impressions act as strong predictors of the overall attitude and acceptability of new technologies, it is important to understand the user experiences of first exposure.

**Methods:**

We conducted semistructured interviews with 18 people 74 years and older who had never used a smart speaker before, investigating the patterns of use, usability issues, and perspectives that older adults have when using a voice assistant for the first time.

**Results:**

The overall first response to a voice assistant was positive, thanks to the simplicity of a speech-based interaction. In particular, a positive and polite response to complete the interaction with a voice assistant was prevalent, such as expressing gratitude or giving feedback about the quality of answers. Two predominant topics of commands made in the first interaction include asking health care–related questions and streaming music. However, most of the follow-up reactions were unfavorable because of the difficulty in constructing a structured sentence for a command; misperceptions about how a voice assistant operates; and concerns about privacy, security, and financial burdens. Overall, a speech-based interaction was perceived to be beneficial owing to its efficiency and convenience, but no other benefits were perceived.

**Conclusions:**

On the basis of the findings, we discuss design implications that can positively influence older adults' first experiences with a voice assistant, including helping better understand how a voice assistant works, incorporating mistakes and common interaction patterns into its design, and providing features tailored to the needs of older adults.

## Introduction

### Background

Recent advances in speech technology and artificial intelligence have made speech a promising form of input modality to interact with personal computing technology. Consequently, a smart speaker with an integrated voice assistant is increasingly available in the market to function as a virtual assistant to perform everyday tasks (eg, Amazon Echo, Google Home). The first commercial version of a smart speaker, Amazon Echo, with an integrated voice assistant, Alexa, was launched in 2015. As of 2019, more than 60 million people in the United States own smart speaker devices, with a 48% annual growth [1]. A voice assistant allows users to perform a range of basic everyday tasks through voice commands, including but not limited to searching for information, streaming music, getting weather and news updates, ordering groceries, and sending and receiving text messages.

As the worldwide population is aging and countries are facing ongoing challenges in caring for their aging population, there has been increased awareness and interest in the potential of smart speaker–based voice assistants to support older adults for their health and independence [2]. Although a voice assistant holds great promise to support older adults through its simple speech-based interaction modality, little is known about how older adults perceive and respond to the idea of talking to a device that does not have a graphical user interface. Researchers have recently begun to investigate older adults' use of a voice assistant, but with few exceptions [3], they produce preliminary or interim reports [2,4,5].

As a first step toward gaining insights into the perspective and use of voice assistants among older adults, this paper investigates novice older users’ first impressions of voice assistants. Since first impressions act as strong predictors of overall attitudes toward new technologies [6], it is important to understand how older adults perceive and respond to a voice assistant when first exposed to it. Through interviews with 18 people aged 74 years and above who had never used a smart speaker, we investigated how older adults interact with a voice assistant in their first use. Specifically, we aimed to answer the following research questions:

1. What usage patterns do older adults have in their first interactions with a voice assistant?
2. What challenges do older adults face when using a voice assistant for the first time?
3. What first impressions do older adults have with a voice assistant?

By answering these questions, we aim to provide insights into the design of a voice assistant that can form a positive first impression among older adults to improve its acceptance and adoption as well as meeting the needs for quality of later life.

The notion of a voice-controlled system has been framed in many different ways, such as a virtual assistant, embodied conversational agent, intelligent personal agent, autonomous agent, or avatar [7]. Throughout this paper, we use the term *voice assistant* to encompass the above terminology and to specifically refer to voice assistants on smart speakers.

### Older Adults and Technology Acceptance

Although new technologies are increasingly being introduced to revolutionize aging in place, the actual acceptance of these technologies is still low [8]. Thus, much effort has been devoted to identifying factors that influence older adults’ use of technology [4,9,10]. The most commonly identified barriers come in the form of aging-related declines [11]. For instance, the small size and low contrast of buttons on a mobile display have a significant negative influence on interaction performance [5]. Another common barrier is related to psychological and mental obstacles, such as negative cognitive perception [3], technophobia [12], and lack of self-efficacy [4]. Older adults especially tend to refuse a new technology due to perceived effort associated with learning [13] or lack of perceived usefulness [14]. As such, several physical and psychological factors were found to have a significant influence on older adults’ technology acceptance.

### Research on Voice Assistants

In recent years, there has been a growing interest within the human-computer interaction community in understanding people’s experiences with voice assistants. Among several, one stream of research has focused on exploring the use of voice assistants in various contexts, such as different locations [15,16] or tasks [17,18]. Another stream of research has focused on investigating factors that constitute an effective conversation with voice assistants, either through personification [8] or using conversational cues [18]. Some researchers, however, argued that user experiences with voice assistants remain disappointing due to a lack of human-like conversational capabilities [19]. Although existing voice assistants are being called *conversational* agents, promising to enable human-like conversation with a device, they are in fact not truly conversational in nature. Instead, simple and constrained request-response structures are the norm, rarely including a realistic dialog [20]. Lastly, research has explored the utility and usability of voice assistants as an assistive technology [21,22]. In particular, researchers are increasingly recognizing the potential that voice assistants can offer in the aging society [5,23], exploring application areas to facilitate voice assistants to support older adults [24,25]. This paper contributes to this emerging body of literature by specifically investigating the first reactions that older adults have to voice assistants.

### Benefits and Challenges of Voice Assistant Use in Older Adult

Voice assistants allow users to interact with it in a universally understood form of interaction modality, speech. Thus, they are deemed to be simple and easy for older adults to use [26]. However, several challenges exist that prevent older adults from interacting with voice assistants [5]. One problem is associated with hearing loss, a common physical complaint in older adults [27]. Because older adults using hearing aids often cannot cope with high levels of ambient noise or have difficulty processing a dialog without contextual information, hearing loss imposes a significant challenge to the use of voice assistants for many of them. Another problem stems from a lack of understanding of the actual expectations and needs of older adults in using voice assistants. Designers are usually considerably younger and may not know about the physical and psychological aspects of aging and have grown up using more advanced technologies than older adults [28]. These problems are important factors to investigate before designing a new system meant to be adaptive and responsive to the perspectives and expectations of older adults. The crucial question is whether a voice assistant is designed to be suitable for older adults. Although researchers have increasingly focused on various usability aspects of voice assistants, relatively less effort has been made to understand them from the perspectives of older adults.

## Methods

### Participants

For participant recruitment, we first contacted a local assisted-living facility located in the greater New York area. We visited the facility and explained the purpose of the study to the manager. Upon their approval, we posted a recruitment flyer in the lobby. Two recruitment criteria were age over 65 years and had no prior experience with a voice assistant. In total, we recruited 18 participants (n=11, 61% females and n=7, 39% males; mean age 79, range 74-91, SD 4.5 years; Table 1). A total of 2 (11%) participants were wearing a hearing aid but did not have any problems with having a conversation. Other than this, no other specific health concerns were reported. Almost all participants (n=16, 89%) were widows or widowers, living in one-person rooms. The other 2 participants were a couple living in a 2-person room. The average length of residency in the facility was 2.3 years (Min=9 months, max=4 years, SD 1.2). All participants said that they were familiar with personal computers, tablets, and smartphones, and 17% (n=3) participants said that they had seen a smart speaker in their children’s homes but had never used it. Seven participants owned a tablet, and all participants reported regularly using a computer for information search and email. We recruited participants from an assisted-living facility for convenience of recruitment. The study protocol was reviewed and approved by the institutional review board.

**Table 1:** Participants’ demographics.

Participant ID	Age (years)	Gender
P1	74	Female
P2	87	Male
P3	76	Female
P4	75	Male
P5	78	Female
P6	83	Male
P7	76	Female
P8	80	Male
P9	75	Female
P10	84	Female
P11	77	Female
P12	78	Female
P13	79	Male
P14	76	Male
P15	79	Female
P16	80	Female
P17	91	Female
P18	82	Male

### Data Collection

We constructed the interview protocol to investigate older adults’ first experiences of and perspectives on the use of voice assistants to answer our research questions. To that end, we created a set of open-ended interview questions with 3 themes: (1) examining first impressions and perceived utility of a voice assistant, (2) identifying common usage patterns of a voice assistant during their first interactions with it, and (3) finding difficulties and challenges participants experience when using a voice assistant. In addition, we collected participants’ basic demographic information, including age, gender, health concerns, and experience with technology.

Participants were randomly assigned to 1 of 3 groups. We conducted an interview with 3 participants in a group so that we could investigate not only participants' experiences with a voice assistant but also their reactions to speech-based interactions initiated and performed by other participants. All participants reported that they knew other participants in a group as they were living in the same facility for years.

To facilitate participants’ interaction with voice assistants, we created 7 decks (categories) of cards, on each of which a voice-command query was written to evoke various functionalities. The categories include getting weather and daily news updates, asking general questions, listening to music, reading a book, playing games, texting and communications, and setting up reminders and alarms. Each deck consisted of 4 to 7 cards depending on the topic of the category. For instance, the deck for weather and news update had 5 cards, which queries include:

- Alexa, what is the weather like today?
- Alexa, will it rain this weekend?
- Alexa, give me a 7-day weather forecast.
- Alexa, what is a headline in the news today?
- Alexa, give me news updates today?

We also created a simple story for each category that illustrates a real-life situation in which the voice-command queries on cards can be used. For instance, for a deck of cards for weather and news updates, we told a story of leaving for a vacation in Milan, when you might want to check the traffic conditions to the airport, the current weather in Milan, and the weather forecast of the day of return.

**Figure 1:**
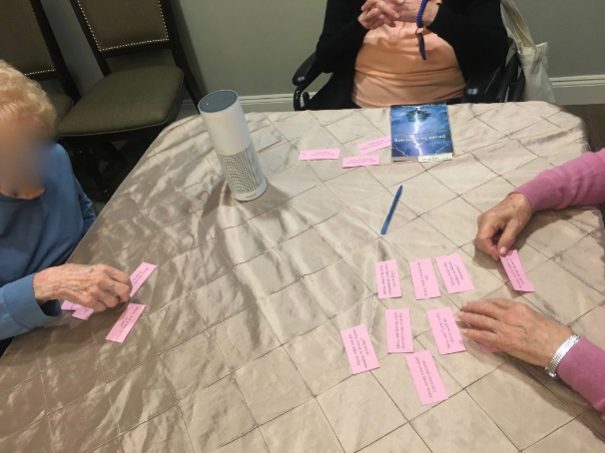
The setting of a semistructured interview with a group of participants.

The interviews were conducted in a meeting room of the facility where a smart speaker (Amazon Echo) was set up (Figure 1). In the interview, we first introduced a smart speaker to participants as “a device that follows your voice command, providing you answers about news, music, weather, and more.” We then demonstrated how to use a voice assistant by asking basic questions about weather and time and by executing simple commands such as streaming music and making a phone call. After this simple introduction, we asked participants to interact freely with the voice assistant as much as they wanted without further training. When participants had no more ideas of what to do with the voice assistant, we then narrated a scenario to inform them of potential use, gave out 1 deck of cards, and asked participants to try out queries written on a card.

Once all participants had enough interaction with a voice assistant and had no more ideas for interaction, they were asked to freely discuss their perspectives on it and its potential utilities for older adults. After completing the interview, participants were compensated monetarily for their participation. Each interview lasted between one and a half and two hours. All interviews were audio-recorded and transcribed.

#### Data Analysis

We analyzed the interview data using thematic analysis to reveal patterns across datasets, through open coding, axial coding, and selective coding [29]. The emerged themes were continuously discussed with another author until data were saturated with recurring themes and no new information was anticipated.

First, we conducted open coding to identify and code concepts significant in the data as abstract representations of events, objects, happenings, actions, etc. The example below explains one participant’s concern about the use of a voice assistant with respect to cost. This response is coded as *Financial concern*:

How much does it cost a month?

P5, Financial concern

Next, we categorized the related concepts created by open coding into conceptual phenomena using axial coding. Phenomena refers to repeated patterns of events, happenings, actions, and interactions that represent people’s responses to problems and situations. For instance, *Misperception* is a phenomenon that represents a participant’s incorrect understanding or interpretation of the operation of voice assistants. During axial coding, the open code *Financial concern* in the example above was categorized as *Misperception* since using a voice assistant does not require a monthly fee unless you subscribe to charged services and as long as you have a wireless internet at home. Lastly, we followed the selective coding process to assemble our conceptual phenomena extracted from axial coding. The goal of this step is to integrate all concepts by building relationships across phenomena.

## Results

### Overview

The overall first response to a voice assistant among our participants was positive, showing a favorable attitude toward the simplicity of a speech-based interaction. However, shortly after interacting with it, they started experiencing difficulty with it, asking a lot of questions and raising concerns about its use. In what follows, we report in detail the findings of our participants' first interactions with a voice assistant by answering our research questions. The main categories of the findings are summarized in Textbox 1.

**Research questions and summary of results.**

R1. What usage patterns do older adults have in their first interactions with a voice assistant?

- Topics of commands made in the first interaction include:
  Asking health care-related questions (91/234, 38.9% interactions);
  Streaming classical music and songs from old days (66/234, 28.2% interactions);
  Asking for directions to or a location of a place (30/234, 12.8% interactions);
- A positive response to complete the interaction with a voice assistant was prevalent, such as expressing gratitude or giving feedback.

R2. What challenges do older adults face when using a voice assistant for the first time?

- Difficulty in constructing a structured sentence for a command
- Misperceptions about how a voice assistant operates
- Concerns about privacy, security, and financial burdens

R3. What first impression do older adults have with a voice assistant?

- A speech-based interaction was perceived beneficial thanks to its efficiency and convenience
- No other benefits were perceived: “It’s not for me.”

### The Usage Patterns of a Voice Assistant

In total, participants made 234 conversational interactions with a voice assistant throughout the interviews. From these interactions, prominent patterns in the initial use of a voice assistant among our participants have emerged in 2 categories: common topics of commands and positive responses to a voice assistant’s answers.

#### Topics of Commands

Since participants had no prior experience using voice assistants, they mostly relied on the cards we provided for the first few interactions. They then came up with their own commands by making variations or extensions of the queries written on cards. Among the varied topics of commands that participants asked a voice assistant, 3 topics were asked most frequently. The topic of the most frequently executed commands was health care-related (91/234, 38.9% interactions), such as body condition, health supplements, and medications (In the excerpts, P# refers to the #th interviewee, VA refers to a voice assistant, Amazon Echo’s Alexa, and *I* refers to an interviewer). Example queries include the following:

Alexa, I got a flu. What kind of medicine should I take?

P4

Alexa, what can I do for arthritis?

P16

Alexa, what does magnesium do for your body?

P18

The next most frequent topic of commands was to stream songs from old days and classical music reminiscent of old times (66/234, 28.2% interactions). The third topic of commands was asking for directions to or a location of a place (30/234, 12.8% interactions). Example queries include the following:

Alexa, play popular songs from the 70s.

P10

Alexa, where is the Veteran's Cemetery in New Jersey?

P2

Other topics of commands include the search for general information, playing audiobooks, and setting up timers and reminders. We expected that participants would ask a voice assistant many questions about the weather forecast and news updates because previous research showed that the most popular smartphone applications among older adults were weather forecasts and news updates [30]. Contrary to our expectations, however, our participants asked a few questions about weather and news updates, except when using queries written on a card, which they expressed little interest in asking. This might be because weather forecasts and news updates are what they would normally check in the morning [30] and thus did not ask in the afternoon when the interviews were held. Alternatively, this might be because they already have their own ways of checking such information (eg, via television news) and thus are less inclined to use a voice assistant to retrieve it. We will discuss the perceived utility of voice assistants in the following section.

#### Positive Response to a Voice Assistant

Interestingly, our participants completed most interactions with a voice assistant by responding back to the answer, either expressing gratitude or giving feedback about the quality of a voice assistant’s answer.

P1: Who is the, Alexa, who is the father of our country?

VA: George Washington was one of the founding fathers of the United States of America and served as the nation's first president.

P1: Good. Thank you.

P9: Alexa, what was the weather in Texas, Austin, yesterday?

VA: Currently in Austin, Texas, it is 73 degrees with clear skies and the sun. You can expect more of the same today with a high of 84 degrees and a low of 61 degrees.

P9: Why do we not move there? I wish we had that weather.

P10: Yeah, I wish that, too.

Lopatovska and Williams [8] have previously framed this type of interaction as personification with mindless politeness that “humans say to each other without meaning anything.” Our observation was different from theirs in that most responses made by our participants had distinct functional purposes. Some responding remarks were used as a cue to finish interacting with a voice assistant as they came back to speak with the interviewer or other participants. Some responses were used as triggers to initiate a follow-up human-human conversation. All these patterns, however, might have resulted from the novelty of using a voice assistant. Because all participants had never used a voice assistant before, they might have expressed their feelings and first impressions through the responding remarks in more extreme attitudes than they would normally do. Alternatively, these patterns might have been shown since the interview was helped as a group with other participants. Thus, further research is needed to investigate how older adults would respond to a voice assistant in an everyday use setting.

### Challenges in Using Voice Assistants

Unlike a common belief, or marketing hype, that it is easy to interact with a voice assistant thanks to its conversational capabilities, most participants experienced difficulty having a *conversation* with it. Prior research has indicated several reasons why people found it difficult to interact with a voice assistant, including the goal of a conversation needing to be highly functional and task-oriented, lack of social aspects of conversation, and a sequential dialog structure of request-response [16,31,32]. Whereas, we found that the challenges our participants experienced were more elementary. Two predominant challenges that emerged from our study include the difficulty in constructing a command sentence and misunderstanding how a voice assistant operates.

#### Difficulty in Constructing a Structured Sentence

To use a voice assistant, a user should first speak a wake word (a word to activate a voice assistant, such as *Alexa* for Amazon Echo or *Hey Google for Google Home*), followed by a concise and definitive sentence for a task. However, many participants kept forgetting to start a command with a wake word or confused the wake word with other similar words throughout the interview.

P15: What would you, what, Alexa, what is a good mystery book to read today? (A voice assistant did not activate and P15 paused for a few seconds...)

P15: Hmm? What’s going on?

P6: What time does the Alaska show on the...

I: You need to start a sentence with “Alexa”

P6: Alaska. No wait. Alexa, what time does the late-night show on the TV tonight start?

VA: Tonight, the late-night talk show starts at...

Even when given a command with a wake word, the voice assistant sometimes did not activate because the command did not have enough pause between a wake word and a following sentence for the voice assistant to capture the wake word or because a wake word was not clearly pronounced. However, using the wrong or no wake word would be an easy fix as users would make fewer mistakes as they continue using a voice assistant. A bigger issue was that many participants struggled to compose a concise command sentence for a task. They often spoke lengthy, unstructured, and descriptive sentences, some of which even included another question within a question. A command with a long sentence resulted in the voice assistant losing track of the voice command and returning to a deactivation mode in the middle of the user speaking. When this happened, participants were not aware that it happened, waited for a response for a few seconds, and became puzzled by why it did not respond to their command. In some other cases, the voice assistant picked up only the first few words of a user’s command due to a pause between words and provided a wrong answer.

P11: Alexa, let me see, I am trying to think of the book. What hat was the title of the book? Ah, can you read the first page of... (Alexa did not activate, and a user paused for a few seconds)

P11: Why is it not answering?

P5: Alexa, what is the status of... the status of the new garbage collection?

VA: Status is a relative position or standing on things, especially persons in the society or a state at a particular time.

P5: It’s giving me a wrong answer.

#### Misperceptions: Operational Inquiries and Concerns

Since participants had no prior experience with a voice assistant, they asked a lot of questions about its use during the interview. Although the interviewer answered all questions, the topics of the questions provided us with insights into how older adults might perceive and expect voice assistants to function. In addition, we found that the underlying reasoning of many such questions stemmed from the misperceptions of how a voice assistant would operate, which resulted in forming negative first impressions.

##### Inquiries About Voice Assistant Operation

The most frequently asked question about voice assistants was how it spontaneously responds to random commands and requests. Most participants did not recognize that a voice assistant retrieves information from the internet. Instead, they supposed that it would retrieve relevant information from the stored local database. This presumption made them expect that they would have to store all the information in a voice assistant before its use. Because of this perception, participants expressed their strong unwillingness to use it or lack of interest in using it due to the perceived effort to store data. In addition, the expectation of having to store all information in a voice assistant aroused privacy and security concerns, which we will discuss in the next section:

[After demonstrating music playing and reading of a voice assistant] Who put the music in it? Where does it get music from?... Does this have a text for a whole book? Where does it get that from?

P13

I think I know how it works or how you make it go. The storage of whatever is in there. That would be a bit of a time-consuming effort to put all of that information on, and whether or not I would want to put certain information on a machine like that. I do not think I would, but you never know.

P4

Once participants were informed that a voice assistant does not require storing data but retrieves information from the internet, they started to explore its capability in various capacities. First, participants interacted with the voice assistant by asking simple factual questions (eg, “who is the 7th president of the United States?”). After receiving a satisfactory answer to such questions, they jumped into asking complicated, and probably impossible to answer, questions (eg, “who will be the next president of the United States?”), to which they received a nonsatisfactory answer. After hopping between asking very simple questions and impossible-to-answer questions several times, participants were dissatisfied with the voice assistants’ capabilities and rejected its adoption.

P11: Alexa, do you think Trump is gonna be re-elected for President?

VA: Sorry, I am not sure how to help with that.

P3: Alexa, can you give me the winning lottery numbers?

VA: Sorry, I am not sure how to help with that.

P3: Of course not.

##### Concerns About Privacy, Security, and Financial Burden

The biggest concern participants expressed was the potential risk associated with privacy and security. Because of the privacy-intrusive potential of a voice assistant’s *always-on* ability to continuously listen to voices in intimate spaces such as the home, privacy concerns about using a voice assistant have been subject to much research over recent years [33,34]. We then found that privacy concerns that our participants had were not only about its capability of *always listening* but also related to their misperception that a voice assistant would store a user’s personal information. Because a voice assistant can respond to a verbal command, participants were concerned that other people could easily retrieve their personal information by talking to a voice assistant:

I think too much information goes into that (a voice assistant) and it worries me. All the information that you put into that machine goes all over the world. That's my concern.

P5

What happens if somebody else asks it (a voice assistant) what's the balance in my checking account? Can you set it up to use a password or a security question so that only you can ask questions to it?

P10

Another prevalent concern was the cost of using a voice assistant. Apart from the cost of the device itself, several participants expected that they would have to pay a monthly service fee to use a voice assistant. Because a voice assistant responds to a user’s question or inquiry, participants considered it as a service to be paid, which they did not want:

All these questions that we asked, you are going to have to pay for, right? How much do you pay? How much does it cost a month?

P3

### Perceived (No) Usefulness of Voice Assistants

Overall, the speech-based interaction modality was well received by all participants. They appreciated the efficiency and convenience of using speech to receive information. After a few interactions, participants started to talk about the potential benefits of voice assistants to help interact with a device without aging-related physical constraints such as vision or mobility:

I used to do a lot more with my eyesight, but now it is the most important thing I have to preserve. I cannot read the screen that is well. Down the road when my eyes fail me as they are slowly doing, I may resort to one of those (a voice assistant) to read things. One of my favorite things is reading, and I would eventually...You know if my eyes go, that's where I might use it (a voice assistant).

P3

I’m not very good at spelling and with computer. It's kind of hard. And I am always afraid that I am going to lose everything by pressing the wrong button. Usually, when I text, I make mistakes. My fingers are too big for the little things, so I hit the wrong buttons usually, so this (a voice assistant) is much better, much faster. I can go about it easier than texting because you do not have to do the typing.

P11

However, most of the rest of the follow-up reactions were not positive, and no other potential benefits were discussed. The most prevalent response to voice assistants was *it’s good, but not for me*. Participants mentioned that a voice assistant might be useful for people other than themselves, such as younger populations with children:

My kids have two children who are really into this (a voice assistant), but it's not for me. I think it's a good thing for somebody who is into various things but for old folks like me we are content with what we are and sometimes ignorance is bliss.

P8

Probably when I was younger with my children and my husband and I had to do everything, you know, when my husband and I were with the children to help their homework and all, that (a voice assistant) would have come in very handy. But at this age there is nothing much to ask.

P17

The fact that many queries written on the cards were simple questions and commands might have influenced this perspective. Answering simple questions and executing simple commands that come up during day-to-day activities in the house is one primary feature of voice assistants. However, we found that this is not what our participants perceived to be the most useful:

I think this device is good in that it can give you the answers to general questions right away. But I’d say there is limited use for elderly people because you really do not get involved in things that you need to ask a bunch of questions about.

P14

It was then not just a first impression that older adults did not see direct benefits of using a voice assistant to them. A recent study showed that the attrition rate of voice assistants among older adults is high primarily due to lack of beneficial uses [3]. This implies that more features tailored to the needs of older adults are required to better assist them with voice assistants. In fact, several features designed specifically for older adults are already available, such as reminding about medications, sending alert messages verbally to their loved ones, and making emergency calls. Although these features were also included in the cards, participants did not find them useful either. Participants said that they were set in their own ways of doing these things and so did not need a new gadget to perform those tasks. For instance, they kept paper diaries for their schedules and used a pillbox to keep track of taking medications. Thus, participants did not perceive the features that are supposed to support older adults useful to them:

I don't have any interest in it (a voice assistant) because I keep my diaries to keep track of things and use a pill box to take medication. And I am content with it. I do not need anything else.

P2

Participants considered a voice assistant to be useful to older adults with aging-related physical constraints who can benefit from hands- and eyes-free interaction. This perspective led them to associate the use of a voice assistant with negative aspects of aging. Several participants mentioned that using voice assistants might make other people think that they were not capable of doing things on their own and needed support. Because aging-related changes are often associated with negative aspects, such as disability, stigma, and dependence, older adults tend to avoid supporting aids and assistive technologies, even though these can be beneficial to them [17,35]. We found a similar pattern in that some participants perceived voice assistants as yet another aid for aging-related declines, associated its use with negative stereotypes of aging, and rejected its adoption. This illustrates that voice assistants may fail regardless of the useful features it offers unless such negative stereotypes are mitigated:

I am healthy enough. My memory is still good enough to remember things. I do not need a device that tells me to remind me of things to do. I do not need a device that tells me to do things. I can still do things on my own.

P10

I think it (a voice assistant) can be useful for those with Alzheimer or old people who cannot move around or cannot do things on their own. I am not like that. I have no problem moving around and doing things. I may use it years later, but not yet.

P15

## Discussion

Our findings revealed the patterns of use, difficulties, and perspectives that older adults might have when they first interact with a voice assistant. On the basis of these findings, we discuss design strategies for voice assistants that would allow older adults to have a positive first impression and to better leverage the capabilities of this technology. The design strategies include helping to better understand how a voice assistant works, incorporating mistakes and common interaction patterns into its design, and providing features tailored to the needs of older adults.

### Help Understand How a Voice Assistant Operates

We found that older adults might have several misperceptions about how a voice assistant operates, which negatively contribute to their perspectives on using it. Three primary misperceptions include perceived efforts to store information before its use, privacy concerns associated with data storage and retrieval, and the cost of its use. In fact, these misperceptions might be universal for all first-time users. However, further considerations should be made to support older adults as they tend to lack self-efficacy about technology and thus experience much more difficulty understanding even basic concepts of new technologies compared with their younger counterparts [14]. Removing these misperceptions and helping them easily understand the basic concepts of how a voice assistant operates would be the first step toward lowering barriers to entry and helping novice older users better explore and facilitate its capabilities.

An immediate and straightforward solution is to provide a voice-based tutorial or educational application to address common misperceptions. Although a user can find out how a voice assistant operates by simply asking relevant questions to it, our participants did not know even what they did not know or what they misunderstood. Therefore, an introductory discourse-based tutorial that explains the basic concepts of how a voice assistant operates might be useful. However, providing a tutorial should be considered a temporary remedy because a well-designed user interface must be intuitive enough for novice users without needing any manuals or instruction.

A long-term solution is to incorporate the basic concepts of how a voice assistant operates into a responding answer, such as the source of information or how to handle personal information, at least in the first few interactions. Although this might increase the number of responses and the total interaction time, successively providing relevant content upon request can reduce mental and temporal burdens on users. In addition, it could be more important for novice users to have an appropriate mental model and understand the system rather than an efficient user experience [32]. For instance, the system can contextualize basic instruction about its operation in the responding answer. The system then completes the response by asking if a user wants to hear more about how it operates. Upon a user’s request, the system can provide more detailed information about the operational mechanism. The system can automatically and gradually reduce the contents related to operating instructions with more usage. The example conversational structure is as follows:

User: Alexa, what is tomorrow’s weather going to be like?

VA: Let me check weather information online. According to AccuWeather, tomorrow’s temperature will be...Do you want to hear more about how I instantly retrieved this information from AccuWeather?

User: Yes, please.

VA: I am connected to the Internet to search for information...

### Incorporate Common Mistakes and Usage Patterns into Voice Interaction

Even though speech is supposed to be easier than typing or clicking, our participants still had difficulties conversing with a voice assistant. We found that a command query should be structured in a particular way that a voice assistant can comprehend (eg, a wake word followed by a concise and definitive sentence for a task after a brief pause) was a primary challenge, making the interaction not truly conversational but rather a series of one-directional comments. Participants especially expressed significant frustration when they realized only after completing a lengthy command that a voice assistant did not activate. One solution to this problem is to separate a wake word and a content sentence in a command structure. That is, instead of speaking the entire sentence for a task at once, a user would first speak a wake word to activate a voice assistant, just like calling someone’s name to draw attention. A voice assistant would then make a simple greeting comment to indicate that it is ready to take commands. This will prevent users from mistake in speaking to a voice assistant when it is not activated. The example conversational structure is as follows:

*User: Alexa*

VA: Hi John, is there anything that I can do for you?

User: Yes, what is tomorrow’s weather going to be like?

VA: The weather tomorrow will be...

One prominent usage pattern we found was that participants completed most interactions with a voice assistant by expressing gratitude to or giving feedback about the quality of a voice assistant’s answer. Although this pattern might be due to the novelty effect, it is still worth considering this usage pattern in the human-agent conversation, since it can provide positive and more conversation-like experiences to novice users. For instance, instead of letting a user complete the interaction, a voice assistant would respond back to the user’s comment and offer suggestions for more features. The example conversational structure is as follows:

User: Alexa, what is tomorrow’s weather going to be like?

VA: The weather tomorrow will be...

User: That’s great. Thanks.

VA: It’s my pleasure. Do you want to check the weather in the next four days?

### Explore Features Tailored to the Needs of Older Adults

The topics of frequently asked commands provide us with a clue to ideas for new features that older adults might find useful. Two topics of primary interest that emerged from our participants’ interactions with a voice assistant include seeking information about health conditions and medication and streaming music from the old days. Thus, enriching the responding contents when executing these commands might give older adults a chance to find more features and functionalities, such as providing relevant or personalized extra information (eg, suggesting more songs to play) or suggesting other features relating to the command (eg, offering local contacts for health care services, providing alternative supplement or medication information).

Although this solution can expose novice older users to a range of new features, a more fundamental question lies in how to positively frame the perspectives that older adults might have about a voice assistant. A positive frame would be to take it from a tool to answer innocuous questions to a useful device that could address the more crucial needs of older adults. Older adults are more likely to adopt a new technology that helps them remain independent, allows them to have control and authority over its features and functions, and does not show signs of aging or frailty [36]. Thus, one solution is to associate the utility of voice assistants with positive aspects and the assets of aging, such as older adults’ skills, knowledge, and resources. For instance, a voice assistant can be a gateway to connect peers within a community (eg, residents of an assisted-living facility) for labor- or information-sharing and social engagement. In this way, the role of a user can be reframed from a passive recipient of services to a proactive actor to provide information and services to others in a community. The mental model of a voice assistant can then be reshaped from a device to support the negative aspects of aging to a pathway to constructively engage in the community. The example conversational structure can be expressed as follows:

User1: Alexa, I am going grocery shopping at 11AM this Saturday and have two seats available to give a ride.

VA: Okay, I will let you know if anyone needs a ride.

User2: Alexa, is anyone going grocery shopping this Saturday? I need a ride.

VA: I found one person. You can get a ride from Jason at 11AM in front of the main entrance…

This solution and all of the previously suggested strategies are just a starting point for further investigation. More research is essential to gain a better understanding of how older adults would naturally interact with a voice assistant and build more naturalistic conversational interfaces to support better human-agent conversations.

### Limitations

Our findings must be evaluated within the context of several limitations. First, our sample size was small, and thus our participant pool may not be representative of a general population. Second, we used convenience sampling for recruitment by recruiting participants from an assisted-living facility, which also runs the risk of compromising generalizability. Selection bias or unmeasured factors (eg, the homogeneity of participant characteristics by living in the same facility) could have influenced the responses during the interviews. Third, our study only investigated the first interactions and experiences that a potential user had when introduced to a voice assistant. Such behaviors might be different from those of users who own a device and use it for their real daily needs. However, we believe that it is important to identify common difficulties that older novice users have in their first interactions with a voice assistant, as it can provide new insights about the design of a device.

### Conclusions

Personal technologies have been considered a breakthrough to tackle challenges associated with aging, and efforts have been made to develop design strategies that meet the needs of the aging population. As part of this effort, this paper explored how older adults would perceive and experience a voice assistant, one fast-growing type of personal technology, when they first interact with it. From interviews with 18 people aged 74 years or above who had never used a voice assistant, we investigated the patterns of use, difficulties, and perspectives that novice older users have when they use a voice assistant for the first time. On the basis of these findings, we discuss design implications that can positively influence older adults' first experiences with a voice assistant, including helping older adults better understand how a voice assistant works, incorporating mistakes and common interaction patterns into its design, and providing features tailored to the needs of older adults. We are hopeful that these findings can be used to expand our knowledge and practices for leveraging emerging personal technologies, a smart speaker–based voice assistant, to support the aging society.
